# The temporal dependence of exploration on neotic style in birds

**DOI:** 10.1038/s41598-017-04751-0

**Published:** 2017-07-06

**Authors:** Mark O’Hara, Berenika Mioduszewska, Auguste von Bayern, Alice Auersperg, Thomas Bugnyar, Anna Wilkinson, Ludwig Huber, Gyula Koppany Gajdon

**Affiliations:** 1 Department of Cognitive Biology, University of Vienna, Wien, Austria; 2 Messerli Research Institute, University of Veterinary Medicine Vienna, Medical University Vienna, University of Vienna, Wien, Austria; 3 Max Planck Institute for Ornithology, Seewiesen, Germany; 4 School of Life Sciences, University of Lincoln, Lincoln, United Kingdom

## Abstract

Exploration (interacting with objects to gain information) and neophobia (avoiding novelty) are considered independent traits shaped by the socio-ecology of a given species. However, in the literature it is often assumed that neophobia inhibits exploration. Here, we investigate how different approaches to novelty (fast or slow) determine the time at which exploration is likely to occur across a number of species. We presented four corvid and five parrot species with a touchscreen discrimination task in which novel stimuli were occasionally interspersed within the familiar training stimuli. We investigated the likelihood that an animal would choose novelty at different stages of its training and found evidence for a shift in the pattern of exploration, depending on neotic style. The findings suggest that faster approaching individuals explored earlier, whilst animals with long initial approach latencies showed similar amounts of exploration but did so later in training. Age rather than species might have influenced the amount of total exploration, with juveniles exploring more than adults. Neotic style varied consistently only for one species and seems to involve a strong individual component, rather than being a purely species-specific trait. This suggests that variation in behavioural phenotypes within a species may be adaptive.

## Introduction

When animals are confronted with novel situations, their behavioural responses are usually determined by fundamental predispositions^1^. One such predisposition is the propensity for exploration, this is classed as any behaviour that serves to gain information but does not satisfy a current physiological need^2^. Responses where exploration is motivated by novelty may be associated with ‘neophilia’^3^ or a preference for novelty^4, 5^, whilst another predisposition, governed by different processes, leads to an avoidance of novel stimuli^6^ and is commonly labelled ‘neophobia’. Exploration is hard to quantify; a variety of approaches have been used^7^, these include measuring the latency to approach a novel object^8^, the manipulation of novel objects^9–16^ and the number of different food items ingested^9, 12^. Conventionally neophobia is measured as the latency to feed in the presence of a novel object^1, 8, 17, 18^, but may also include other measures (such as a bias towards familiar food or places)^19^. It is generally assumed that neophobia is associated with a reduction in exploration^20^ and the terms exploration and neophilia have been used interchangeably in the literature^19^. This stems from the fact that most experimental procedures only assessed neophilia in single trials (see refs 5 and 7 for a review), leading to the assumption that a fast approach to novel items is associated with exploration of these and thus neophilia and exploration are often considered synonymously^1, 21^. These ambiguities have led to inconsistencies and confusion concerning the relationship between exploration, neophilia and neophobia, with the latter two also subsumed under the term ‘neotic’ responses in earlier literature^4, 5, 19, 22^. To keep neophilia and exploration separated and ensure clarity we heron use the term ‘neotic style’ to address the final motivation (as the result of competing neophobic and neophilic tendencies) to approach novel stimuli (measurable as fast or slow approaches), whereas we reserve the term ‘exploration’ for interactions with such novel items.

Recent research has suggested that exploration and neophobia are controlled by fundamentally different processes^1, 5, 7, 21^ and, as such, different predictions can be made regarding the environmental factors that promote different levels of exploration and neophobia in birds. A recent model^1^, has suggested that feeding ecology and habitat complexity are key factors that determine exploration levels, whereas neophobia is impacted by riskiness of foraging and interspecific competition. Thus, behavioural predispositions are not shaped by ultimate pressures alone^15, 19^, individual experience can impact upon the functional expression of exploration and neotic styles, resulting in differences between individuals within a species^23–27^. Individual rank within dominance hierarchies has been shown to influence approaches to novelty. In some species higher ranking individuals exhibit lower latencies to feed in close proximity to a novel object^28^, whereas in other species subordinate individuals may be less neophobic towards novel food^18, 29^ though this relationship can also be dependent on the method of assessment^18^. Developmental conditions have further been reported to effect neophobia, whereby individuals raised in more enriched environments showed lower levels of neophobia in later life^17, 26, 30, 31^. Even within an individual, levels of exploration may vary, this is most prominently observed during ontogeny^9, 12, 19, 32^. It has been proposed that young individuals have sensitive periods in which they exhibit high levels of exploratory behaviour, supporting the hypothesis that exploratory behaviour yields the acquisition of information^9, 19^. However, exploration and play during juvenile life can be difficult to tease apart and may often also be confused with each other^33^.

Exploration, neophilia and neophobia are generally thought to be important factors affecting problem solving capacities and complex behaviour such as tool use^5, 10, 14, 16, 19, 33–43^. However, recent work has suggested that decreased neophobia had little influence over problem solving abilities^5^. Furthermore, evidence to support the idea that there will be increased innovation rates in neophilic animals due to the propensity to exploit novel resources is far from conclusive^5^. Rather, it may be a characteristic of widely distributed, generalist, families, such as corvids and parrots^5, 39^. As they are predominantly opportunistic feeders, that occur in different habitats^1^ they represent ideal taxa to investigate the interdependence between neotic style and exploration.

**Figure 1:**
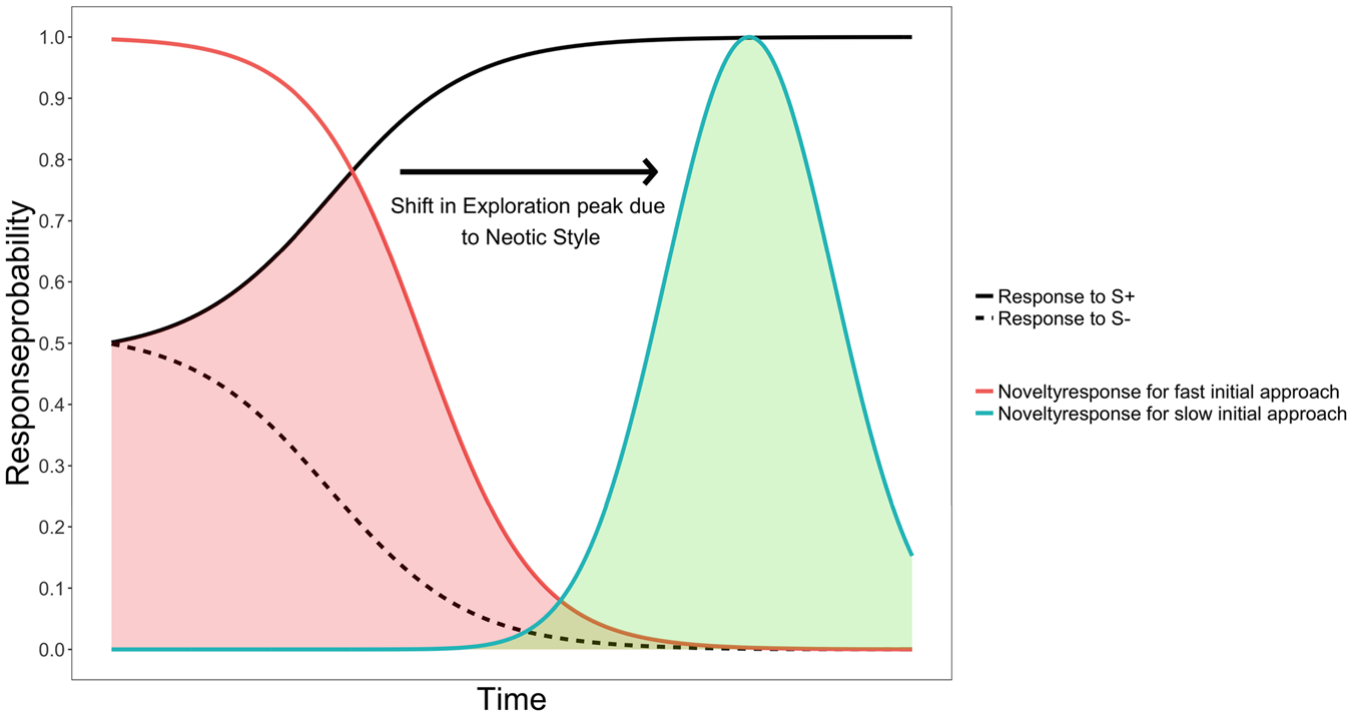
Theoretical model illustrating the shift in exploration-probability dependent on neotic style; solid- and perforated black lines indicate assumed responses to baseline stimuli (S+ and S−) throughout the course of learning; coloured lines represent proposed total novelty response probability for individuals approaching novelty fast or slow over time, with assumed normal distribution, if novel stimuli are non-rewarded; shaded areas denote corrected total novelty responses; hence, we assume the total amount of corrected novelty responses (shaded area) as well as the height of the peak to be dependent on how explorative an individual is and its general learning capacity, whereas the location of the peak on the time axis to be defined by what neotic style an individual pursues.

We hypothesise that neotic style does not affect the amount of exploration, but rather effects the point at which exploration is expressed and predict that the timing of exploration behaviour will shift, depending on the neotic style of the individual being tested (see Fig. 1). Thus, individuals quickly approaching novelty should peak in their propensity to explore at the early stages of a task, whereas individuals that approach novelty slowly would be more likely to select novelty once they have had substantial experience with the task. Further, we hypothesise that individual level selection (e.g. position in social structure) may play a pronounced role in the development of neotic style on top of selective forces acting at a species level (e.g. predation risk). Therefore, neotic style might be subject to pronounced individual variation, on top of ecologically grounded species-specific predispositions. If neotic style is based solely on species level propensities, one would expect to find consistent behavioural patterns within a species. Alternatively, if neotic style is expressed mainly on an individual level we expect to find large variation in responses within a species, and limited or no species-specific differences. Finally, as exploration is important for the acquisition of information, one might expect more exploration in the early stages of life, where most learning occurs^9^. Following this trend in the literature^9, 13^, we predict that young individuals might exhibit generally more explorative behaviours than older individuals.

In order to test these hypotheses, we presented birds with a two-alternative forced choice procedure on a touchscreen. This apparatus was chosen as it allowed both the direct comparison across species and the opportunity to present an almost unlimited number of highly controlled novel stimuli. To assess changes in response to novelty during the task, the unrewarded stimulus was replaced with a novel one in two of the 16 trials in each session, this occurred over 16 sessions. Thus, the novel stimulus was presented against a previously reinforced stimulus, making the choice of novelty an uncertain one.

To discern whether neotic styles are controlled by species- or individual-level traits, we compared the performance of nine different species from two taxa, corvids and parrots. These species were selected to represent different taxa from specific ecological backgrounds which we predict would impact upon neotic style. Species endemic to, or originating from, islands are thought to face less predation pressure and therefore the costs for neophilia would decrease. Thus, a neotic style with low delay to approach novelty could be expected in kea (*Nestor notabilis*), Goffin’s cockatoos (*Cacatua goffiniana*), vasa parrots (*Coracopsis vasa*) and New Caledonian crows (*Corvus moneduloides*) in contrast to carrion crows (Corvus corone), raven (Corvus corax), jackdaws (*Corvus monedula*), eclectus parrots (*Eclectus roratus*) and African grey parrots (*Psittacus erithacus*), which all are rather widely distributed, and have increased predation risk^1^.

As a proxy for neotic style, we calculated delta latencies using the differences in response latency between the first trial of the final session and the first trial of the first session (after habituation and pre-training to touch the screen). Individuals were attributed to four types of neotic style (Very Fast Approach, Fast Approach, Slow Approach and Very Slow Approach) using quartiles of the total range of these latencies. Exploration was defined as choosing the novel stimulus over the rewarded one in the test trials. These responses were corrected for weak association with the rewarded stimulus by multiplying the number of novelty choices with the observed probability of choosing the rewarded stimulus in baseline trials of each session. The number of overall responses to the unrewarded stimulus throughout the task was used as an inverse measure of learning.

## Results

### Factors Influencing the Approach of Novelty - Neotic Style

**Figure 2:**
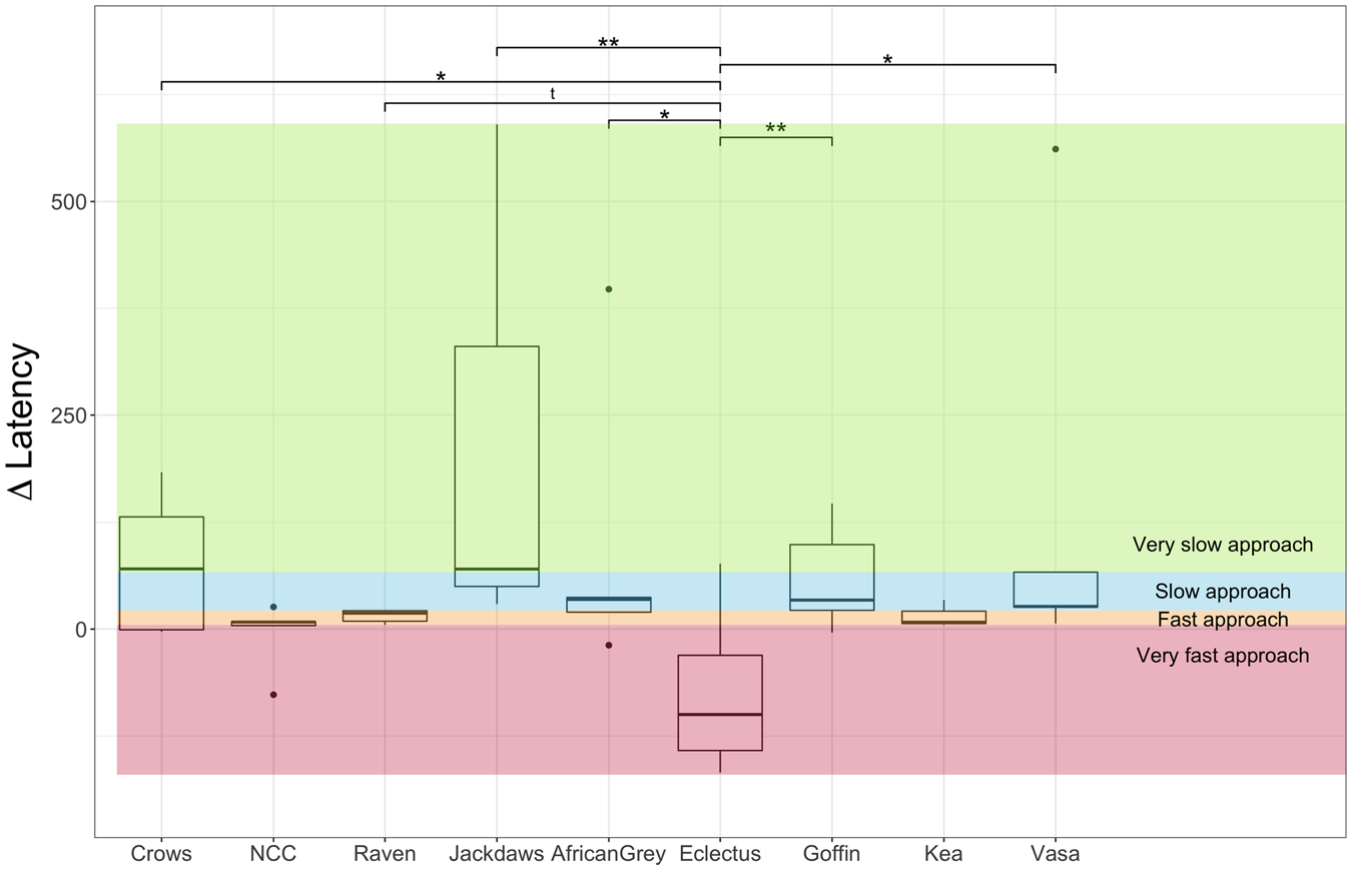
Delta latencies (approach latency in the last, non-consecutive session subtracted from the approach time in the first trial of session one) for all species; bold horizontal lines indicate median values, boxes span the first to third quartiles and whiskers represent 95% confidence intervals; horizontal lines indicate species comparisons; Significance codes: ‘***’ for *p* < 0.001, ‘**’ for *p* < 0.01, ‘*’ for *p* < 0.05, ‘t’ for *p* < 0.1 (alpha adjusted for multiple comparisons).

General linear models revealed neither an effect of corrected novelty responses (GLM: F_1,33_ < 0.01, p = 0.96), nor of the responses to unrewarded baseline stimuli (GLM: F_1,34_ = 0.01, p = 0. 93) or sex (GLM: F_1,35_ = 0.41, p = 0.53) on the delta approach latencies. Age had no significant effect (GLM: F_2,41_ = 1.08, p = 0.35) in a parallel model and was discarded in favour of species in the model structure, this showed a significant effect (GLM: F_8,36_ = 2.65, p = 0.021), indicating that eclectus parrots exhibited significantly lower delta latencies than African grey parrots (β = −92.49, SE = 1.15, p = 0.027), crows (β = −85.75, SE = 29.83, p = 0.029), Goffin’s cockatoos (β = −108.21, SE = 28.84, p = 0.006), jackdaws (β = −124.52, SE = 35.97, p = 0.01) and vasa parrots (β = −103.10, SE = 31.15, p = 0.011). However, no other significant species differences were observed (see Fig. 2).

### Overall Novelty Responses – Total Exploration

**Figure 3:**
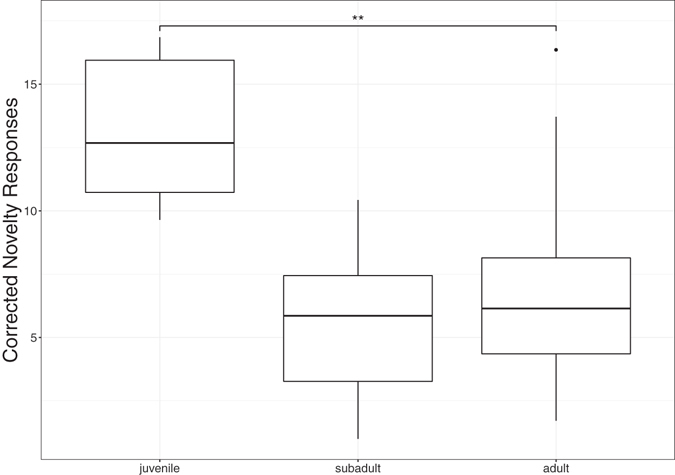
Age differences in corrected novelty responses; bold horizontal lines indicate median values, boxes span the first to third quartiles and whiskers represent 95% confidence intervals; horizontal lines indicate species comparisons; Significance codes: ‘***’ for *p* < 0.001, ‘**’ for *p* < 0.01, ‘*’ for *p* < 0.05, ‘t’ for *p* < 0.1 (alpha adjusted for multiple comparisons).

The total amount of explorative responses over the whole experiment was effected by species (GLM F_8,39_ = 2.82, p = 0.014). However, this effect is driven by the large number of novelty responses exhibited by juvenile carrion crows who chose novel stimuli more often than five other species; no other species differences were observed (see Supplementary Fig. S1 and Table S1 for detailed contrast results). As age and species were confounding factors for carrion crows, we discarded species in favour of age as a fixed factor in a separate model. Age yielded a significant effect (GLM: F_2,45_ = 5.66, p = 0.006), with juveniles exploring significantly more than adults (β = 0.50, SE = 0.15, p = 0.003), but not more than subadults (β = 0.34, SE = 0.24, p = 0.21). No difference was found between adults and subadults (β = 0.16, SE = 0.21, p = 0.46; see Fig. 3). In both parallel models (one including species, the other age), the amount of incorrect first choices had a significant effect on explorative responses (GLM: F_1,45_ = 7.32, p = 0.01) reflecting a positive relationship between exploration and choice of the unrewarded stimulus in baseline trials (β = 0.008, SE = 0.003, p = 0.013; see Fig. S2). No influence of sex (GLM: F_1,36_ = 0.10, p = 0.75), distribution of species (GLM: F_1,37_ = 0.72, p = 0.40) or neotic style (GLM F_3,38_ = 0.90, p = 0.45) was found.

### Novelty Responses Over Time – The Temporal Expression of Exploration

**Figure 4:**
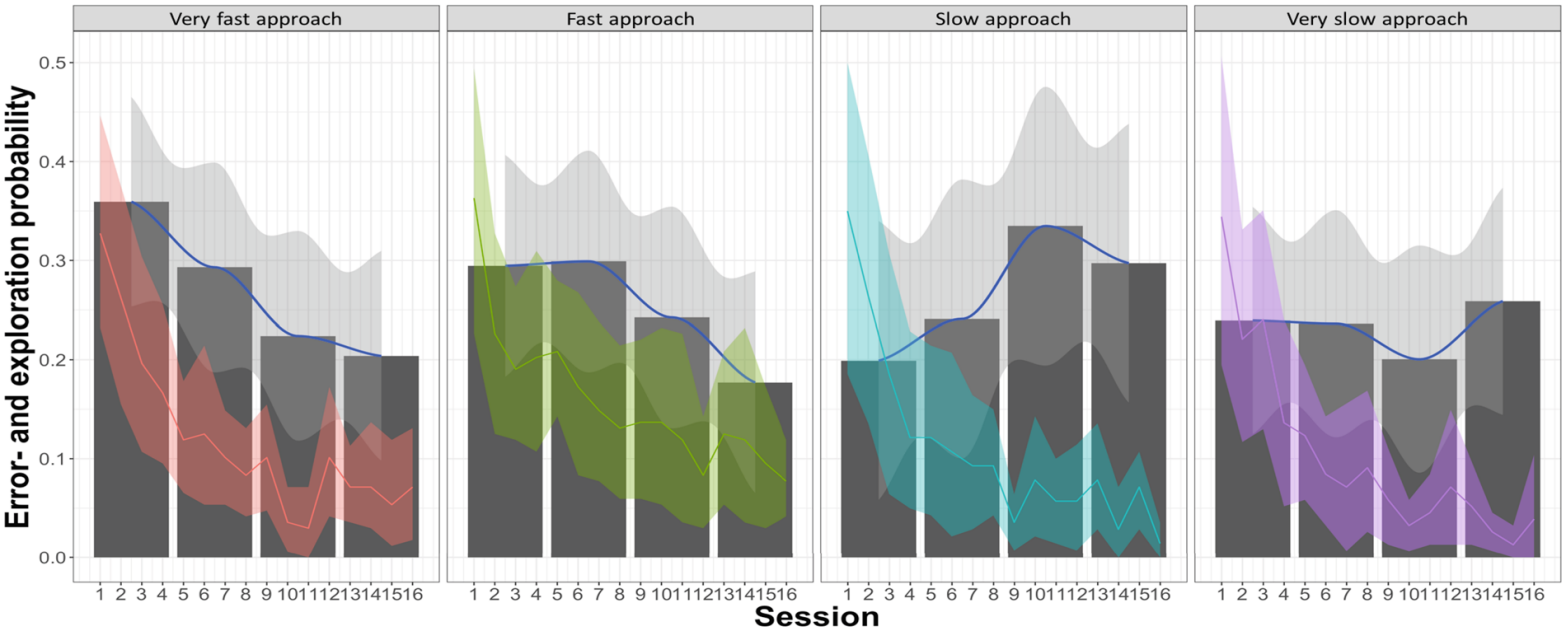
Bars show mean corrected probability to commit novelty responses, in each of the four task quarters for different groups of neotic responses; blue line indicates the smoothed slope by local polynomial regression fitting (locally weighted scatterplot smoothing-loess); the coloured lines indicate the probability to respond to the unrewarded baseline stimuli; shaded areas represent 95% confidence intervals.

To investigate the time-dependent development of exploration, data for all the sessions were pooled and then split into four blocks (each containing 25% of the data for that individual). Analysis of explorative responses over blocks revealed a significant interaction of species and block (GLMM: $\chi$ ^2^(8) = 18.56, p = 0.02). But as this effect rests mainly on juvenile carrion crows exhibiting a significantly different slope of novelty responses over time than jackdaws (GLMM: β = −1.21, SE = 0.40, p = 0.045), kea (GLMM: β = −1.37, SE = 0.41, p = 0.032) and a tendency to differ from vasa parrots (GLMM: β = −1.07, SE = 0.37, p = 0.05) and African Grey parrots (GLMM: β = −0.91, SE = 0.35, p = 0.09; see Supplementary Fig. S4 and Table S2 for additional information, as well as Fig. S5 for performance by session), we discarded species in favour of investigating the interaction between age and block as a fixed factor. While the model did not reveal a significant interaction between choosing the unrewarded baseline stimuli and block (GLMM: $\chi$ ^2^(1) = 0.09, p = 0.77), or an interaction of sex and block (GLMM: $\chi$ ^2^(1) = 0.20, p = 0.66), a significant effect of block was found for the interaction with age (GLMM: $\chi$ ^2^(2) = 6.45, p = 0.04), indicating that juveniles tend to show different temporal exploration patterns than adults (GLMM: β = 0.49, SE = 0.20, p = 0.058). However, no differences in slopes over blocks were found between subadults and juveniles (GLMM: β = −0.12, SE = 0.34, p = 0.72), nor adults and subadults (GLMM: β = 0.37, SE = 0.32, p = 0.35). Examination of the impact of neotic style on the block in which exploration was highest revealed a shift from the first block for very fast approaching (individuals with low delta latencies), to the last block for very slowly approaching individuals (with high delta latencies; see Fig. 4). This is statistically supported by the significant interaction of neotic style by block affecting the slope of novelty responses throughout the task (GLMM: $\chi$ ^2^(3) = 11.28, p < 0.01). Model contrasts revealed that slopes between very fast approaching and very slowly approaching individuals (GLMM: β = 0.55, SE = 0.24, p = 0.058), as well as fast and slowly approaching individuals (GLMM: β = 0.57, SE = 0.26, p = 0.058) bordered significance and differed significantly between fast and very slowly approaching individuals (GLMM: β = 0.81, SE = 0.26, p = 0.015). Other comparisons were not significant (see Supplementary Table S3 for detailed contrasts).

## Discussion

Our results reveal that neotic style does not impact upon the amount of exploration observed but rather effects the time at which it takes place. Very fast approaching individuals exhibited most novelty responses in the early trials of discrimination learning, but individuals with increased latencies to approach, exhibited a peak in their exploration which was shifted towards the later stages of the task. This result suggests that neophobic individuals do not necessarily explore less, but rather do so once they have habituated to a situation. Corvids and parrots, have been proposed to be amongst the most neophilic and explorative bird species^1, 5^ however, as this work generally investigates the behaviour on a group level, individuals that are fast to approach novelty can potentially influence the group data. Thus the inter-individual differences observed here are likely to be a general phenomenon and a noteworthy addition to the general findings of species level differences observed in the literature^1, 8^.

**Figure 5:**
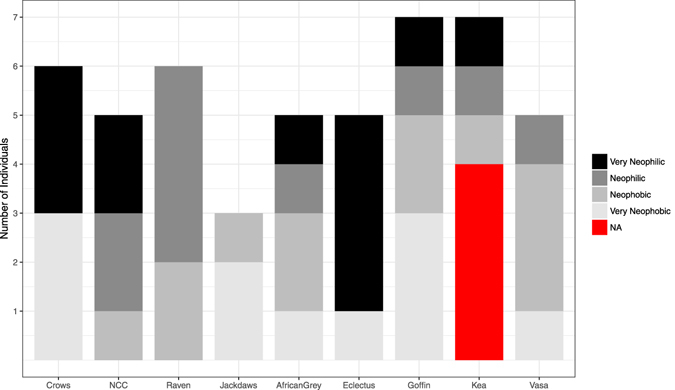
Number of individuals per species participating in the task. Shading indicates quartile latencies to respond to either stimulus in the first trial of the task after the touchscreen has been associated with a food reward: Black represents very fast approaching individuals (responding below 5.00 seconds); dark grey indicates fast approaching individuals (responses made between 5.00 and 21.97 seconds); slowly approaching individuals (responding within 21.97 and 66.87 seconds) were assigned medium grey; light grey shows very slowly approaching individuals (requiring more than 66.87 seconds to interact with the stimuli); the red bar indicates missing values of four individuals of kea for which no latencies are available.

The results reveal little effect of species or distribution patterns in terms of exploration. A clear difference in exploration over time can only be observed in juvenile crows (see Fig. S4 and Table S2), suggesting that age rather than species underlies this effect. While the finding that crows responded most strongly to novel stimuli is in line with ecological predictors, such as largest distribution patterns and omnivorous diet^1^, this species effect is heavily confounded by age. A recent study with the same individuals, that has shown that explorative tendencies deteriorate as age increases^12^. Further, neotic style, did not differ consistently between species, except for eclectus parrots who generally exhibited relatively low delta latencies to approach the novel stimuli (see Figs 2 and 5). This suggests that, rather than being driven at a species level, and being determined by potential risks connected with foraging^1, 19^, neotic style is more likely to result from an interaction between the social structure of the species, individual position in the social hierarchy and individual level experience^1, 13, 18^. These factors are likely to promote the large individual differences observed in this study.

It has recently been suggested that dominance hierarchies will influence neophobia, with higher ranking individuals expressing lower levels of neophobia in some species^28^, but not in others^20, 29^. Future comparative studies should investigate the assumption that inter-individual variation in neotic style would be less pronounced in solitary species. In contrast, gregarious species may have increased variation due to intraspecific competition, thus the largest range of variability in neotic style should be found in despotic social structures with strong competition and strict rank hierarchies. This interrelation might potentially also explain the differences that we observed regarding neotic style and age. Young individuals often are granted certain ‘liberties’ and social tolerance before being integrated into socially structured hierarchies^44, 45^, especially among kin^46^. If the social structure is related to the establishment of different neotic styles, one may expect a potential shift from neophilic to neophobic behaviour to depend on the formation of these rank hierarchies. Indeed, in ravens rank hierarchies have been reported to be established early in development^46^ (in month 4–5 after fledging) and the shift in neotic style occurs before the subadult stage^12^ (18^th^ month). However, further studies testing these assumptions directly may yield valuable insights into the interplay between neotic style, age and social structure of different species.

This study is the first to show the temporal effect that different neotic styles have on exploration, while neophilic individuals explore earlier, neophobic animals do not explore less, but rather express the behaviour later, after familiarisation with the context. The behavioural variations found in the species tested here are suggestive of a general phenomenon and contribute to a more coherent understanding of the inter-relationship between neotic style and exploration. Being neophilic does not imply an individual never stops exploring and conversely, neophobia does not exclude exploration. Thus, our findings allow for a more accurate interpretation of behaviour and the processes which control responses to changes in the environment.

## Methods

### Study subjects

Ravens (*Corvus corax*) and carrion crows (*Corvus corone*) were group-housed at the Haidlhof Research Station (University of Vienna and the University of Veterinary Medicine Vienna, Austria). Hand-reared ravens and carrion crows were kept in adjacent aviaries (each 10 m × 12 m × 4 m) and tested in neighbouring, visually isolated compartments (3 m × 4 m × 4 m). Both groups were fed a diet of meat, pasta, curd cheese and bread twice per day with water for drinking provided ad libitum. Four male and two female ravens were tested, whereas two males and four female carrion crows participated in the study. All individuals were juveniles within their first summer after hatching.

Five New Caledonian crows (*Corvus moneduloides*) and three jackdaws (*Corvus monedula*) were tested at the Avian Cognition Research Station of the University of Oxford, U.K., hosted by and associated with the Max Planck Institute for Ornithology, Germany. The New Caledonian crows were wild-caught and kept in groups of three (a breeding pair and a subadult female) and two (a breeding pair) individuals within aviaries (3 m × 5 m × 2.5 m) accompanied by heated indoor roosting places (1 m × 3 m × 2 m) which also served as testing compartments. Diet consisted of meat, curd cheese, oats, cereal, fruit and cat food, as well as fresh water, which was provided once in the morning and available ad libitum. Jackdaws (two adult males and one adult female) were hand-raised and housed in a large outdoor group aviary (15 m × 9 m × 2.8 m) including testing compartments (2 m × 3 m × 2.8 m). Diet was the same as for the New Caledonian crows.

Kea (*Nestor notabilis*) were hand- and parent-raised and housed at the Konrad Lorenz Institute for Comparative Ethology in Vienna, Austria, in a large (15 m × 10 m × 4 m) enriched group aviary. They received a diet of fruit, vegetable, protein and seed twice a day as well as daily fresh water was provided ad libitum. Seven keas were tested, four males (two adult and two subadult) and three females (one adult and two subadult).

Five individuals of each, vasa (*Coracopsis vasa*), eclectus (*Eclectus roratus*) and African Grey parrots (*Psittacus erithacus*) were tested at the Lincolnshire Wildlife Park in collaboration with the University of Lincoln, U.K. Each species was housed in an aviary consisting of a heated indoor compartment (5 m × 2 m × 2 m) including a testing chamber (1 m × 1.5 m × 2 m) and enriched outdoor compartment (5 m × 4 m × 3 m). The groups were provided a diet of vegetables, eggs, fruit and seeds throughout day and water was provided in the outdoor compartment ad libitum. In the group of eclectus, two individuals were females and three were males, whereas among African Grey parrots (two females, three males) and vasa parrots (one female, four males). Sex was assessed by morphological traits (size, colouration, behaviour). Exact ages were unknown, but all individuals had been kept at the park for longer than two years and therefore were considered as adults.

Goffin’s cockatoos (*Cacatua goffiniana*) were all hand-raised and housed at the Goffin Lab in lower Austria). Seven individuals were tested, including one female and six males. One male subject was subadult and six subjects were adult.

### Ethical Statement

All subjects that participated in reported experiments were housed in accordance with the Austrian Federal Act on the Protection of Animals (Animal Protection Act—TSchG, BGBl. I Nr.118/2004). Furthermore, as the present study was strictly non-invasive and based on behavioural observations, all experiments are classified as non-animal experiments in accordance with the Austrian Animal Experiments Act (§ 2, Federal Law Gazette No. 501/1989).

### Apparatus

The study was conducted on a touchscreen computer which was an adapted mobile version of the operant conditioning system described by Steurer *et al*.^47^. The mobile version combined a CPU (based on a Schneider A4F® minicomputer (http://www.mappit.de) with Mini-ITX main board (VIA EPIA1 M10000, with 1-GHz CPU, 2 × USB, 1 × LAN 10/100 Mbit, sound, and VGA on board), 512 MB DDR RAM, a 40-GB 2.5-in. hard disc) and feeding system in one sealable cube (385 mm × 500 mm × 610 mm) with touch sensitive screen and a reward tray (60 mm × 60 mm × 20 mm) located in the front and flap on the back allowing access to a second screen, keyboard and mouse. The feeding wheel was attached behind the touch sensitive screen and would rotate one reservoir at a time, thus releasing a reward below the screen into the small tray, whenever a stimulus with positive contingency was touched. The screen was a 15-inch XGA colour TFTLCD Module (Model G150XG01 by AU Optronics Corp., Taiwan; http://www.auo.com), with a display area of 304 mm × 228 mm (381-mm diagonal) and a resolution of 1.024 × 768 pixels. A 15-inch IR “CarrollTouch” touchframe (Model D87587-001, 15 in., without a filter) by Elo (Menlo Park, CA; http://www.elotouch.com) was attached on top of the screen for detecting responses on the screen. The opening for delivering the reward was located centrally 80 mm below the lower edge of the screen. The software program used for cognitive testing was CognitionLab (version 1.9; see ref. 47 for detailed description).

### Procedure

All individuals participating in this study were naïve to any touchscreen computer setup. Therefore, a habituation and a two-choice discrimination pre-training phase preceded the actual task. Rewards consisted of 1/16^th^ of Frolic™ for raven and carrion crows, one mealworm for jackdaws and New Caledonian crows, 1/4^th^ of a peanut seed for kea, vasa, eclectus and African Grey parrots and 1/6^th^ of a cashew nut for Goffin’s cockatoos. Each individual was tested separately from the group, except for the initial habituation phase were a group approach was employed. Only individuals which successfully concluded the habituation phase progressed to all the further tasks.

#### Discrimination task

After completion of the pre-training and discrimination training (see supplementary information for details of these procedures), each individual was presented with a randomly assigned novel baseline stimulus pair (S_1_+ and S_1_−), containing more visually complex (differing in colour and shape) stimuli (see Supplementary Fig. S6 for an exemplary stimulus set and information on how the stimuli were generated). As in discrimination training, each session consisted of 16 training trials but also included two novelty trials. These occurred within the first half of each session (pseudo-randomly at trial 5, 6 or 7) and in the second half of each session (pseudo-randomly at trial 11, 12 or 13). These novelty trials were either *identical* novelty trials, in which the rewarded S_1_+ remained the same and only S_1_− was replaced by a new unrewarded stimulus (S_2_−), or a *similar* novelty trials, in which S_1_− was replaced by a novel stimulus (S_3_−), as well as the positive stimulus S_1_+ being replaced by a slightly different one (slight variation in colour and shape; S_n_+). A new negative novel stimulus was displayed in every novelty trial throughout the task, while S_n_+ differed from each other in the first eight sessions and the same set of S_n_+ were then used respectively in the last eight sessions again. As in the discrimination training, pecking on an unrewarded stimulus resulted in a CT until S_1_+ was pecked.

Correct first choices (CFCs), CTs, pecks on screen (POS), response latencies, and stimulus positions for each trial were recorded.

### Analysis

Pearson’s product moment correlation was used to assess the relation of total number of novelty responses in similar and identical novelty trials. As the novelty responses in these trials were positively correlated (Pearson correlation: *t* = 5.77; n = 48, *p* < 0.001, *r* = 0.677; see Supplementary Fig. S7), responses to novel stimuli were pooled as a measure of total novelty responses. In order to correct for novelty responses solely based on a weak association with the S_1_+, responses towards novel stimuli were multiplied by the observed probability to choose the rewarded stimulus in baseline trials of each session. Learning performance was assessed as the number of incorrect choices committed in baseline trials (see Supplementary Material for effects on learning performance).

Individual neotic style was assessed by the latency to the first response in the discrimination task subtracted from the latency to approach the apparatus in the first trial of the last (non-consecutive) session. This measure is equivalent with conventional measures of neophobia where latencies to feed next to a novel item were recorded and corrected for general latency to approach food^5, 7^. In this case individuals, had associated the touchscreen with food rewards and novel items were represented by the two unknown stimuli (S_1_+ and S_1_−). However, this procedure may also be considered as a measure of neophilia as the motivation to approach could have been intrinsically linked to the stimuli themselves^7^.

Due to technical issues, latencies were not assessed for four keas and therefore latencies for these individuals were treated as missing variables. Linear models were used to investigate potential effects of species, exploration (as the amount of novel stimuli chosen in novelty trials corrected by the error probability in baseline trials), incorrect first choices in baseline trials (as a measure of learning), age and sex on the delta latencies (time to respond to any stimulus in the first trial minus latency to respond in the first trial of the last non-consecutive session). The total range of these delta latencies were then divided into quartiles, determining four levels of neotic style, in which individuals would be grouped: ‘Very Fast Approach’ < 5 sec. < ‘Fast Approach’ < 21.97 sec. < ‘Slow Approach’ < 66.87 sec. < ‘Very Slow Approach’. (see Figs 2 and 5 for distribution of different groups according to their response latencies in each species).

General linear models, assuming quasi Poisson distribution, were used to investigate the effect of exploration (as the amount of novel items chosen in novelty trials corrected by the error probability in baseline trials), neotic style, species, sex and age on the learning ability, as the amount of first incorrect choices in baseline trials. General linear models, with assumed quasi Poisson distribution, were employed to examine the effect of sex, age, species, learning ability and neotic style as fixed factors on exploration in the task.

To investigate the temporal effect of neotic style, age and species on exploration in this task the sixteen sessions were separated into “blocks”, each containing four sessions. Linear mixed models were then employed to test interactions of block with neotic style, with age and with species. Individuals were introduced as random factor to account for repeated measures.

As age and species were confounded variables (as ravens and crows consisted solely of juveniles, but no other species included juveniles) we ran each model twice including either age or species as a fixed factor and report the results for the more sensible model structure. Whenever species was removed as a fixed effect we introduced distribution as a fixed effect to contrast island living with widely distributed species. Normality of residuals was tested using Shapiro-Wilk test for normality and confirmed visually, while the assumption of homoscedasticity was tested for using the studentized Breusch-Pagan test, where appropriate. Models were fitted by creating a full model (including all fixed effects) and stepwise single term reduction using the “drop1”-function^48^. Test statistics given refer to changes in fit relative to the original. Statistical analysis was carried out in R^49^ version 3.2.3. Models were calculated using the lme4-package^50^ and graphical representation of results was created using the package ggplot2^51^. Alpha levels were set to 0.05, factor level contrasts were set manually, employing the “glht” function of the package “multcomp”^52^ for multiple comparisons of independent variables. P-values were adjusted for multiple testing employing the false discovery rate correction^53, 54^ at group level and all statistical tests were conducted two-sided.

## Electronic supplementary material

Supplementary Information
