# Supplementary material for: The temporal dependence of exploration on neotic style in birds
Source: Sci Rep. 2017 Jul 6;7:4742. doi: 10.1038/s41598-017-04751-0 (PMC5500574; doi:10.1038/s41598-017-04751-0)
Supplement: Supplementary file 1 — Supplementary Information [file 41598_2017_4751_MOESM1_ESM.pdf]

# SUPPLEMENTARY INFORMATION

## The temporal dependence of exploration on neotic style in birds

Mark O'Hara*[1,2], Berenika Mioduszewska[3], Auguste von Bayern[3], Alice Auersperg[2], Thomas Bugnyar[1], Anna Wilkinson[4], Ludwig Huber[2], Gyula Koppany Gajdon[2]

1 Department of Cognitive Biology, University of Vienna, Austria

2 Messerli Research Institute, University of Veterinary Medicine Vienna, Medical University Vienna, University of Vienna, Austria

3 Max Planck Institute for Ornithology, Seewiesen, Germany

4 School of Life Sciences, College of Science, University of Lincoln, United Kingdom

* Corresponding author

Email: mark.ohara@univie.ac.at

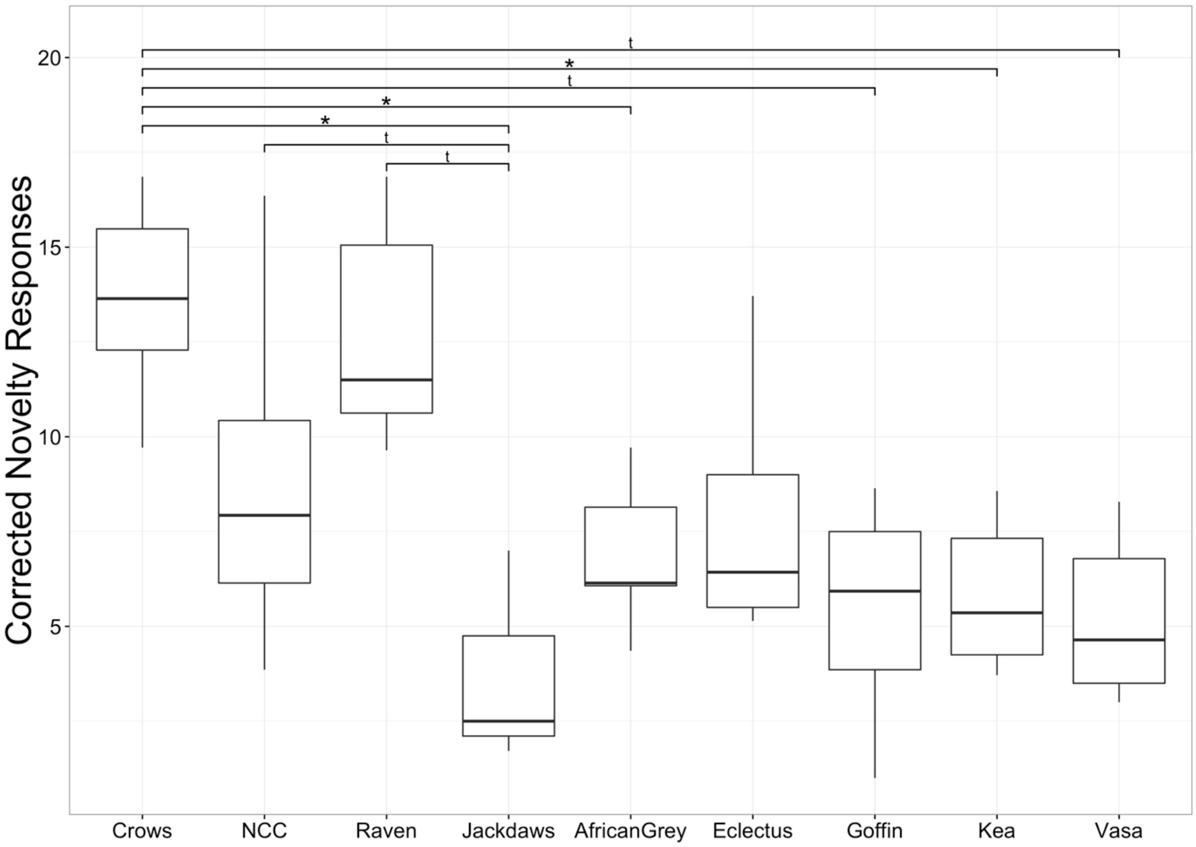


**Figure S1**. Boxplots of corrected novelty responses for all species; bold horizontal lines indicate median values, boxes span the first to third quartiles and whiskers represent 95% confidence intervals; horizontal lines indicate age group comparisons; Significance codes: '***' for $p < 0.001$, '**' for $p < 0.01$, ‘*’ for $p < 0.05$, 't' for $p < 0.1$ (alpha adjusted for multiple comparisons; see Table S1 for detailed test statistics).

**Table S1.** Multiple comparisons of means for novelty responses, corrected for association strength in each session, of all species. P values are adjusted for multiple comparisons.

Model: *Corrected Novelty Responses ~ Sum of errors + Species*

Significance codes: '***' for $p < 0.001$; '**' for $p < 0.01$; '*' for $p < 0.05$; '.' for $p < 0.1$

| | Estimate | Std. Error | t value | P | Sig. |
|---|---|---|---|---|---|
| AfricanGrey vs Crows | 0.628 | 0.206 | 3.049 | 0.028 | * |
| AfricanGrey vs Eclectus | 0.219 | 0.236 | 0.929 | 0.482 | |
| AfricanGrey vs Goffin | 0.004 | 0.254 | 0.015 | 0.988 | |
| AfricanGrey vs Jackdaws | -0.482 | 0.350 | -1.377 | 0.310 | |
| AfricanGrey vs Kea | -0.059 | 0.237 | -0.249 | 0.904 | |
| AfricanGrey vs NCC | 0.359 | 0.231 | 1.551 | 0.257 | |
| AfricanGrey vs Raven | 0.416 | 0.223 | 1.868 | 0.185 | |
| AfricanGrey vs Vasa | -0.042 | 0.276 | -0.153 | 0.930 | |
| Crows vs Eclectus | -0.408 | 0.202 | -2.022 | 0.155 | |
| Crows vs Goffin | -0.624 | 0.229 | -2.722 | 0.058 | . |
| Crows vs Jackdaws | -1.109 | 0.329 | -3.374 | 0.014 | * |
| Crows vs Kea | -0.687 | 0.204 | -3.364 | 0.014 | * |
| Crows vs NCC | -0.269 | 0.197 | -1.365 | 0.310 | |
| Crows vs Raven | -0.212 | 0.171 | -1.239 | 0.337 | |
| Crows vs Vasa | -0.670 | 0.253 | -2.643 | 0.059 | . |
| Eclectus vs Goffin | -0.216 | 0.237 | -0.912 | 0.482 | |
| Eclectus vs Jackdaws | -0.701 | 0.341 | -2.057 | 0.155 | |
| Eclectus vs Kea | -0.278 | 0.225 | -1.239 | 0.337 | |
| Eclectus vs NCC | 0.139 | 0.220 | 0.635 | 0.653 | |
| Eclectus vs Raven | 0.196 | 0.227 | 0.866 | 0.497 | |
| Eclectus vs Vasa | -0.262 | 0.261 | -1.005 | 0.454 | |
| Goffin vs Jackdaws | -0.485 | 0.345 | -1.408 | 0.310 | |
| Goffin vs Kea | -0.063 | 0.232 | -0.270 | 0.904 | |
| Goffin vs NCC | 0.355 | 0.229 | 1.553 | 0.257 | |
| Goffin vs Raven | 0.412 | 0.266 | 1.550 | 0.257 | |
| Goffin vs Vasa | -0.046 | 0.255 | -0.181 | 0.930 | |
| Jackdaws vs Kea | 0.423 | 0.339 | 1.245 | 0.337 | |
| Jackdaws vs NCC | 0.840 | 0.336 | 2.498 | 0.064 | . |
| Jackdaws vs Raven | 0.898 | 0.348 | 2.580 | 0.059 | . |
| Jackdaws vs Vasa | 0.439 | 0.362 | 1.215 | 0.337 | |
| Kea vs NCC | 0.418 | 0.218 | 1.916 | 0.181 | |
| Kea vs Raven | 0.475 | 0.232 | 2.046 | 0.155 | |
| Kea vs Vasa | 0.017 | 0.257 | 0.065 | 0.976 | |
| NCC vs Raven | 0.057 | 0.224 | 0.255 | 0.904 | |
| NCC vs Vasa | -0.401 | 0.253 | -1.583 | 0.257 | |
| Raven vs Vasa | -0.458 | 0.287 | -1.599 | 0.257 | |

**Factors Effecting Learning**

Responses to unrewarded baseline stimuli were significantly affected by the amount of corrected novelty responses (GLM: $F_{1,39} = 8.24$, $p = 0.007$), indicating that individuals that responded more often to novel stimuli chose the unrewarded stimulus more often in baseline trials ($\beta = 0.08$, SE = 0.03, $p = 0.039$; see Fig. S2). Additionally, species had an effect on the amount of incorrect first choices in baseline trials (GLM: $F_{8,39} = 2.83$, $p = 0.014$), with Goffin's cockatoos choosing the unrewarded stimulus significantly less often than African Grey parrots ($\beta = -1.26$, SE = 0.38, $p = 0.032$) and ravens ($\beta = 1.27$, SE = 0.40, $p = 0.041$). Also vasa parrots exhibited less incorrect first choices than African Grey parrots ($\beta = -1.21$, SE = 0.43, $p = 0.048$) and tended to perform better than ravens ($\beta = -1.21$, SE = 0.45, $p = 0.056$; see Fig. S3). Other factors, such as neotic style (GLM: $F_{3,31} = 0.11$, $p = 0.95$) or sex (GLM: $F_{1,38} = 1.80$, $p = 0.19$), did not affect learning performance. Age revealed no significant effect in a parallel model (GLM: $F_{2,45} = 1.05$, $p = 0.36$) and was discarded in favour of a model structure including species as a fixed effect.

While noetic style did not seem to influence learning ability, we did find a positive correlation of the amount of novelty responses with the number of unrewarded choices in baseline trials. The fact that overall greater exploration indicated more responses to unrewarded stimuli may reflect general impaired associative skills, especially in young birds. As we controlled for associative strength of the rewarded stimulus in instances of novelty trials, and age did not affect learning ability, this explanation seems unlikely. Another possibility would be that more explorative individuals are less coherent in their choices and frequently 'revisit' the known unrewarded stimuli. Interestingly, this result stands in stark contrast to previous studies that found a positive effect of exploration on discrimination learning[1].

---

[1] Guillette, L. M., Hahn, A. H., Hoeschele, M., Przyslupski, A. M. & Sturdy, C. B. Individual differences in learning speed, performance accuracy and exploratory behaviour in black-capped chickadees. *Anim. Cogn.* 165–178 (2014). doi:10.1007/s10071-014-0787-3

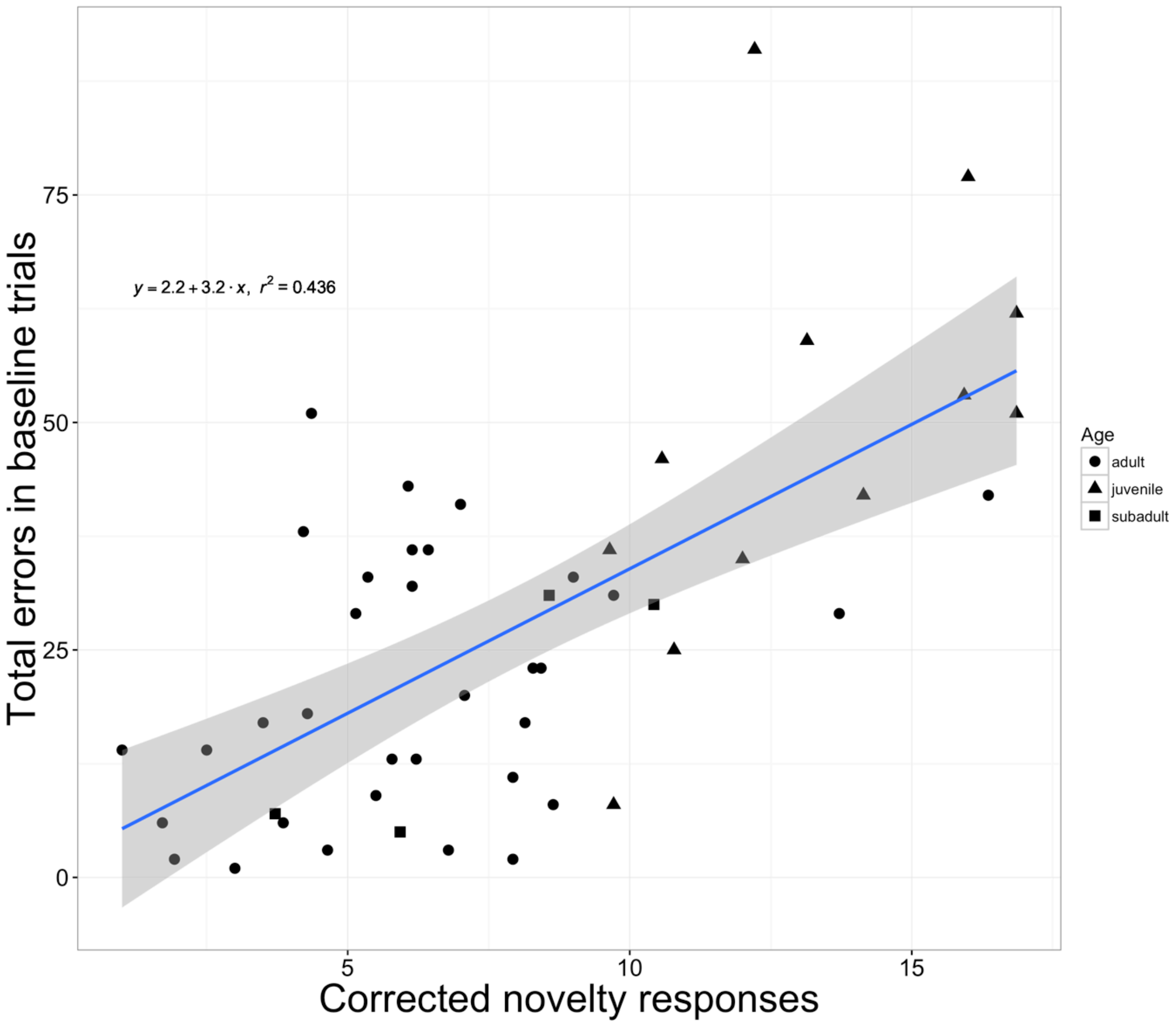


**Figure S2**. The relationship of learning performance, determined by the number of incorrect first choices in baseline trials, and total amount of corrected novelty responses in the task for individual birds; shape of points represents different age groups; blue line indicates assumed linear fit; grey area denotes 95% confidence interval.

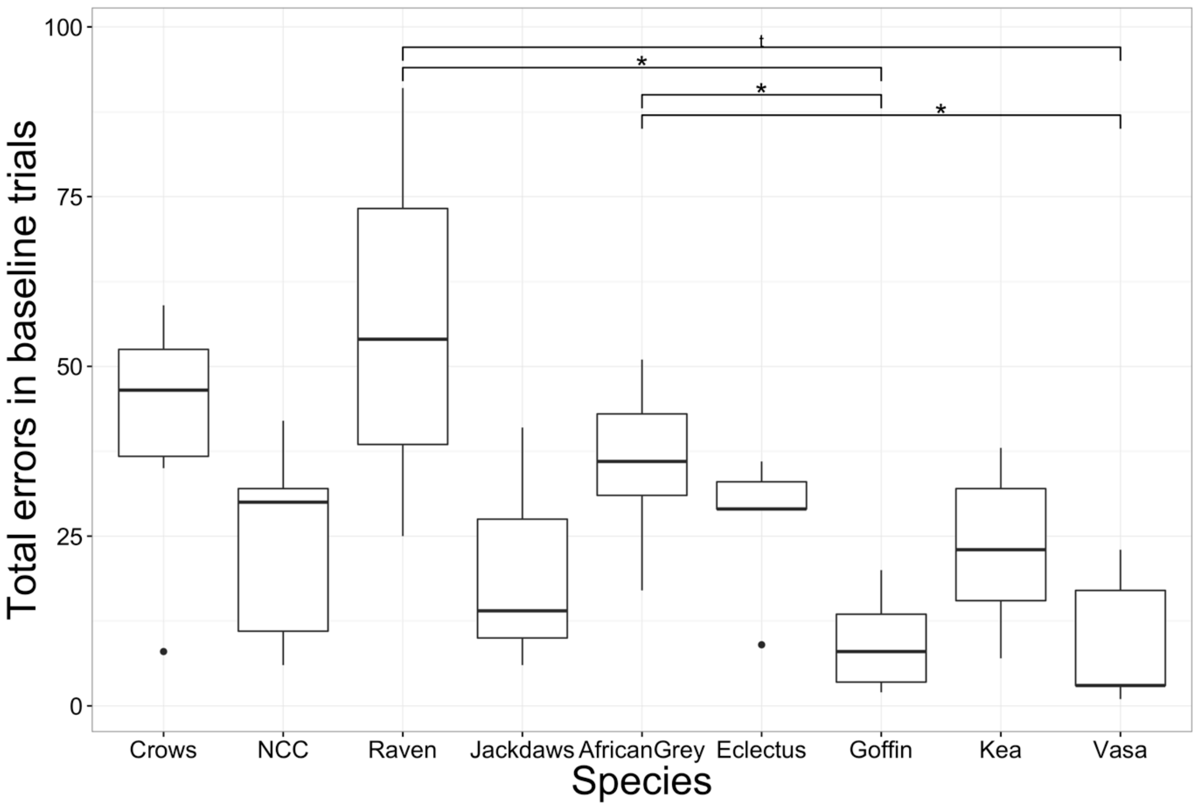


**Figure S3**. Boxplots of total amount of novelty responses, corrected for associative strength with the rewarded stimulus in each session, for all species; bold horizontal lines indicate median values, boxes span the first to third quartiles and whiskers represent 95% confidence intervals; horizontal lines indicate species comparisons; Significance codes: '***' for $p < 0.001$, '**' for $p < 0.01$, '*' for $p < 0.05$, 't' for $p < 0.1$ (alpha adjusted for multiple comparisons).

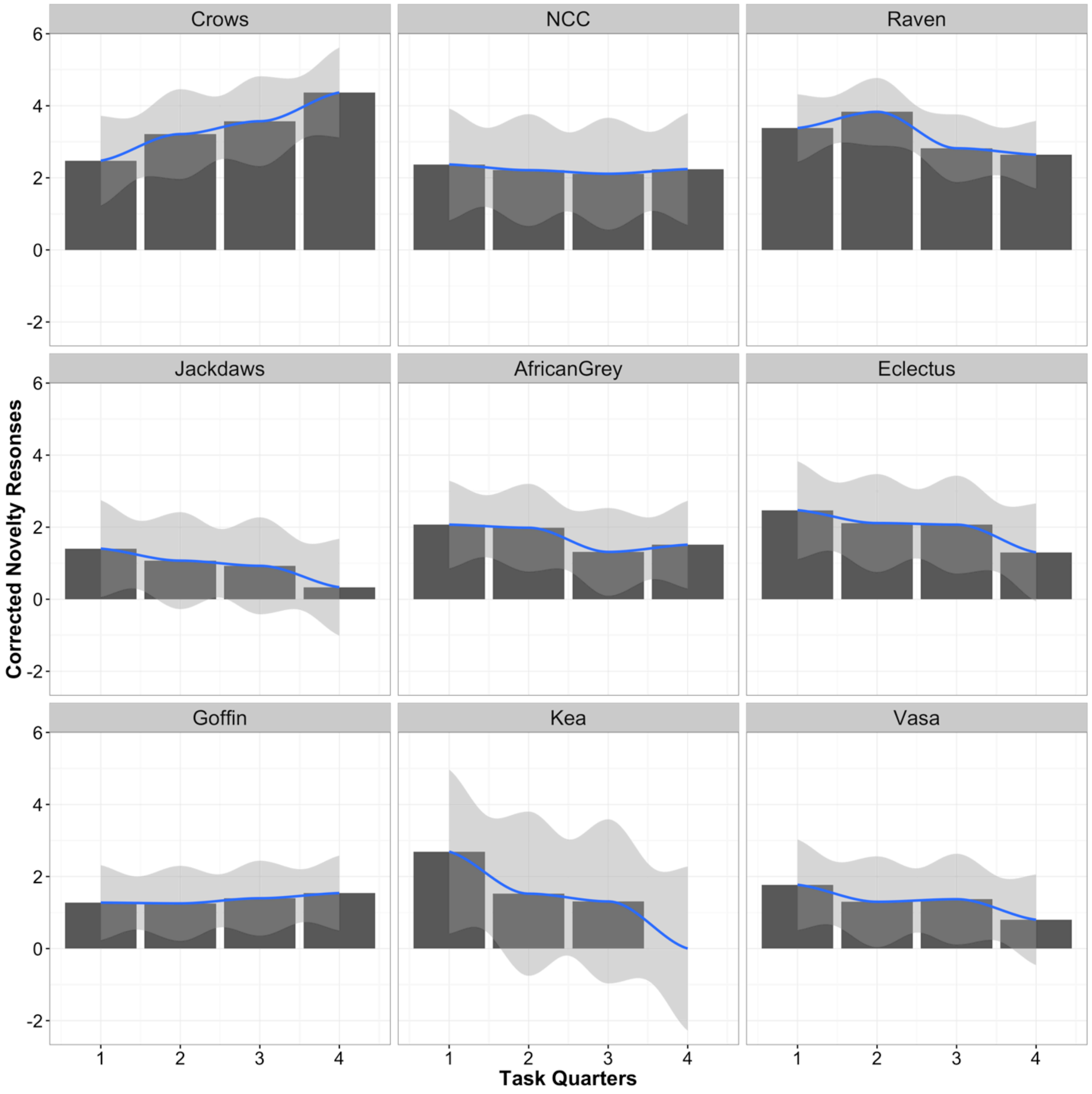


**Figure S4**. Bars show mean expressed exploration, as corrected novelty responses, in each of the four task quarters for different species; blue line indicates the smoothed slope by local polynomial regression fitting (locally weighted scatterplot smoothing-loess); shaded areas represent 95% confidence intervals.

**Table S2.** Multiple comparisons of slopes for the novelty responses, corrected for association strength in task quarters of all species. P values are adjusted for multiple comparisons. Significance codes: '***' for $p < 0.001$; '**' for $p < 0.01$; '*' for $p < 0.05$; '.' for $p < 0.1$

Model: *Corrected Novelty Responses in TaskQuarters ~ NeoticStyle*TaskQuarters + Species*TaskQuarters + (1 + TaskQuarter | Individual)*

| | **Estimate** | **Std. Error** | **t value** | **P** | **Sig.** |
|---|---|---|---|---|---|
| AfricanGrey vs Crows | 0.910 | 0.353 | 2.576 | 0.090 | . |
| AfricanGrey vs Eclectus | 0.117 | 0.376 | 0.310 | 0.801 | |
| AfricanGrey vs Goffin | 0.270 | 0.321 | 0.839 | 0.640 | |
| AfricanGrey vs Jackdaws | -0.305 | 0.410 | -0.745 | 0.640 | |
| AfricanGrey vs Kea | -0.459 | 0.399 | -1.149 | 0.531 | |
| AfricanGrey vs NCC | 0.389 | 0.351 | 1.108 | 0.533 | |
| AfricanGrey vs Raven | 0.017 | 0.348 | 0.049 | 0.961 | |
| AfricanGrey vs Vasa | -0.160 | 0.347 | -0.461 | 0.796 | |
| Crows vs Eclectus | -0.793 | 0.337 | -2.353 | 0.128 | |
| Crows vs Goffin | -0.640 | 0.318 | -2.012 | 0.199 | |
| Crows vs Jackdaws | -1.215 | 0.402 | -3.025 | 0.045 | * |
| Crows vs Kea | -1.368 | 0.412 | -3.322 | 0.032 | * |
| Crows vs NCC | -0.521 | 0.359 | -1.451 | 0.407 | |
| Crows vs Raven | -0.892 | 0.388 | -2.302 | 0.128 | |
| Crows vs Vasa | -1.070 | 0.374 | -2.863 | 0.050 | . |
| Eclectus vs Goffin | 0.153 | 0.351 | 0.436 | 0.796 | |
| Eclectus vs Jackdaws | -0.422 | 0.437 | -0.965 | 0.602 | |
| Eclectus vs Kea | -0.575 | 0.424 | -1.358 | 0.449 | |
| Eclectus vs NCC | 0.272 | 0.370 | 0.735 | 0.640 | |
| Eclectus vs Raven | -0.099 | 0.408 | -0.244 | 0.831 | |
| Eclectus vs Vasa | -0.277 | 0.401 | -0.690 | 0.654 | |
| Goffin vs Jackdaws | -0.575 | 0.379 | -1.518 | 0.389 | |
| Goffin vs Kea | -0.728 | 0.387 | -1.884 | 0.238 | |
| Goffin vs NCC | 0.119 | 0.336 | 0.355 | 0.801 | |
| Goffin vs Raven | -0.252 | 0.337 | -0.748 | 0.640 | |
| Goffin vs Vasa | -0.430 | 0.328 | -1.310 | 0.457 | |
| Jackdaws vs Kea | -0.153 | 0.470 | -0.327 | 0.801 | |
| Jackdaws vs NCC | 0.694 | 0.431 | 1.612 | 0.385 | |
| Jackdaws vs Raven | 0.323 | 0.432 | 0.747 | 0.640 | |
| Jackdaws vs Vasa | 0.145 | 0.412 | 0.352 | 0.801 | |
| Kea vs NCC | 0.847 | 0.397 | 2.135 | 0.168 | |
| Kea vs Raven | 0.476 | 0.396 | 1.203 | 0.515 | |
| Kea vs Vasa | 0.299 | 0.406 | 0.736 | 0.640 | |
| NCC vs Raven | -0.371 | 0.345 | -1.077 | 0.533 | |
| NCC vs Vasa | -0.549 | 0.362 | -1.515 | 0.389 | |
| Raven vs Vasa | -0.177 | 0.346 | -0.512 | 0.783 | |

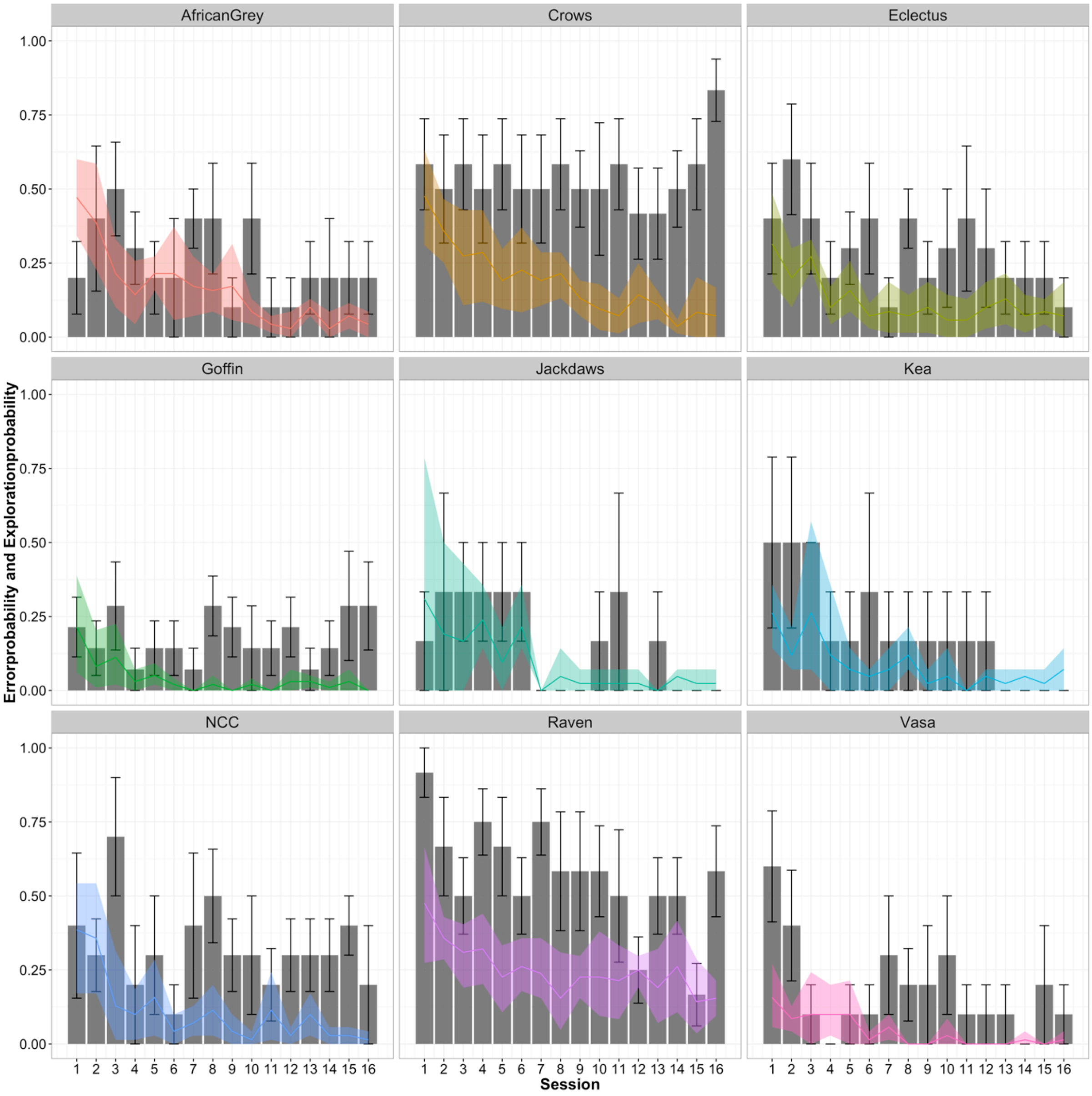


**Figure S5**. Bars show the mean observed corrected probability to respond to novel stimuli in each session, with whiskers representing SE; coloured lines indicate learning curves as mean probability of committing an error in baseline trials, with shaded areas representing 95% confidence intervals for different species.

**Table S3.** Multiple comparisons of slopes for the novelty responses, corrected for association strength in task quarters of all species. P values are adjusted for multiple comparisons. Significance codes: '***' for $p < 0.001$; '**' for $p < 0.01$; '*' for $p < 0.05$; '.' for $p < 0.1$

Model: *Corrected Novelty Responses in TaskQuarters ~ NeoticStyle*TaskQuarters + Age*TaskQuarters + (1 + TaskQuarter | Individual)*

| | Estimate | Std. Error | t value | P | Sig. |
|---|---|---|---|---|---|
| Very Fast Approach vs Fast Approach | -0.261 | 0.244 | 1.070 | 0.355 | |
| Very Fast Approach vs Slow Approach | 0.314 | 0.238 | 1.316 | 0.339 | |
| Very Fast Approach vs Very Slow Approach | 0.552 | 0.242 | 2.282 | 0.058 | . |
| Fast Approach vs Slow Approach | 0.575 | 0.258 | 2.233 | 0.058 | . |
| Fast Approach vs Very Slow Approach | 0.813 | 0.258 | 3.151 | 0.015 | * |
| Slow Approach vs Very Slow Approach | 0.238 | 0.237 | 1.004 | 0.355 | |
| Adult vs Juvenile | 0.489 | 0.200 | 2.446 | 0.058 | . |
| Subadult vs Juvenile | -0.119 | 0.335 | -0.356 | 0.722 | |
| Adult vs Subadult | 0.370 | 0.323 | 1.147 | 0.355 | |

**Stimulus Generation**

For this study a total of 48 stimuli were generated and divided into 12 simple habituation stimuli, 18 straight line stimuli, 18 curved stimuli (see Fig. S6 for examples). For the discrimination task each individual was randomly assigned a specific baseline pair of a positive (rewarded) and negative (non rewarded) stimulus. The baseline pairs contained one straight lined and one curved stimulus. Additionally, for each positive Stimulus 16 similar stimuli were created (differing slightly in colour and shape from the original positive stimulus). The colours of the stimuli were assigned randomly from a scale, differing in their RGB-scale by 20 steps from one colour to the next, but not in brightness. First the positive stimulus was randomly assigned to a colour, saving five colours above and below on the scale for the similar stimuli. Then the negative stimulus was assigned the most distant colour from the positive stimulus colour, excluding the six colours above and below the negative one. The remaining colours were then randomly assigned to the remaining stimuli.

**Procedure**

*Pre-training*

In order to pre-train the animals to use the touchscreen computer they were presented with a simple geometric figure (circle, triangle, square or star) that was centred on the screen. Individuals were shaped to reliably touched the stimulus and received the reward by moving the mouse cursor around the stimulus or in case of limited approach the screen was introduced in a group setting.

After this initial phase and once the stimulus was touched reliably by the animals, the stimulus was then presented in a random position. This phase consisted of two sessions with 35 trials each. Inter-trial-intervals (ITI) were set to one second, during this time the screen was blank.

*Discrimination training*

In this phase, each individual was given one additional stimulus, which was not rewarded when responded to. Additionally, a correction inter-trial-interval (CITI) of 1.3 seconds was introduced. The two stimuli were presented next to each other, with the rewarded stimulus ($S_0$+) displayed randomly on either the right or left side on a horizontal axis on the screen. The height of the stimuli was adapted

for each species to ensure the stimuli would appear at head height. If the subjects pecked the unrewarded stimulus ($S_0$-) the birds received a correction trial (CT), which was repeated until the $S_0$+ was selected. Pecking a stimulus resulted in differential acoustic feedback being played (depending on the stimulus that was being pecked).

Discrimination training consisted of two sessions with 16 trials each. The computer program recorded the retention latency (latency from onset of the trial until a peck occurred), correct first choices and number of correction trials, as well as pecks on the screen in other locations than where the stimuli were.

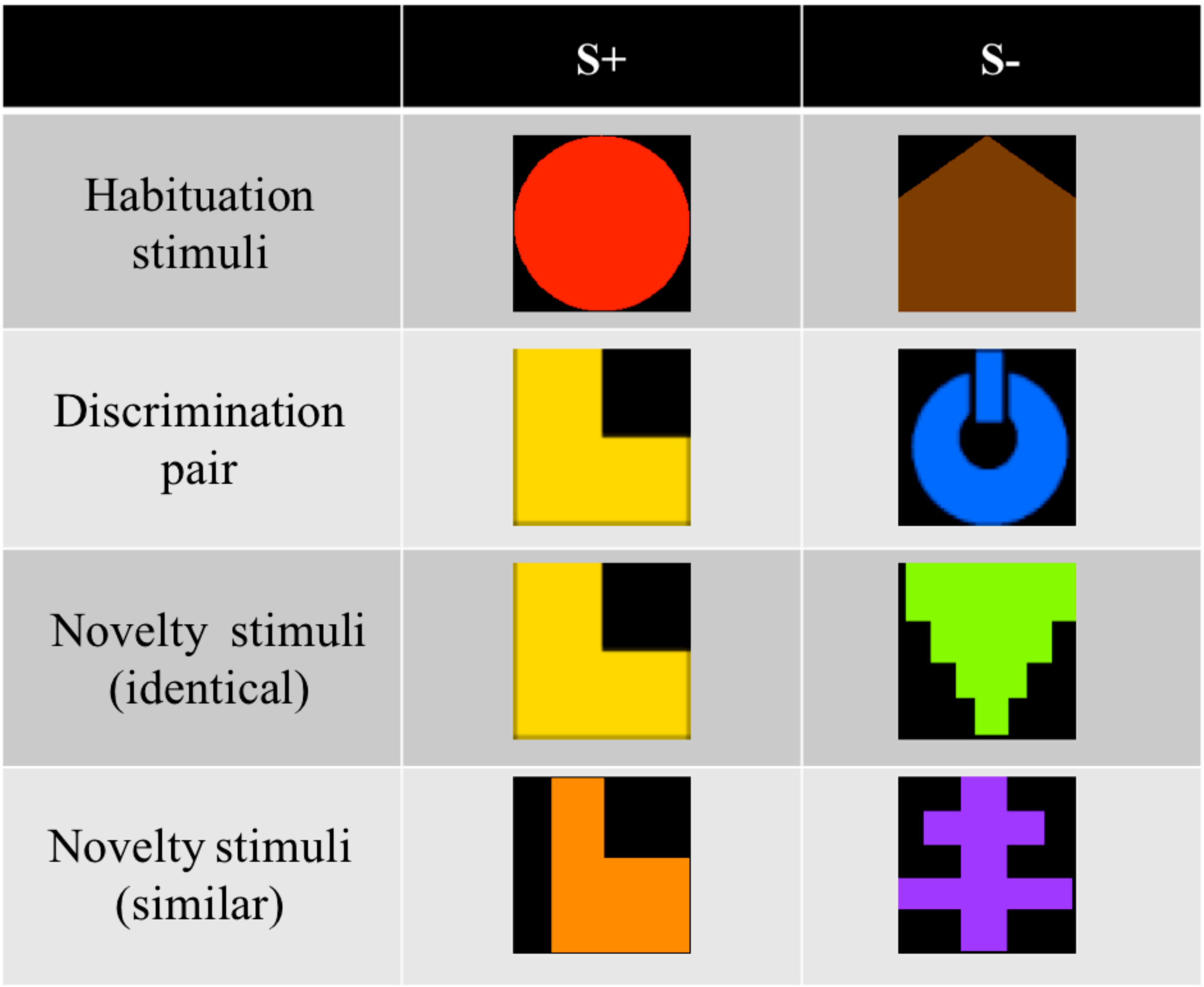


**Figure S6**. Examples of generated stimuli used throughout the task. Habituation stimuli were used during habituating birds to the setup, Discrimination pair exemplifies stimuli used for the main discrimination task, Novelty stimuli were used during novelty test trials with the rewarded stimulus remaining the same as in the basic discrimination task (identical) or being slightly altered in colour and shape (similar); S+ indicates the rewarded stimuli, S- lists the unrewarded stimuli.

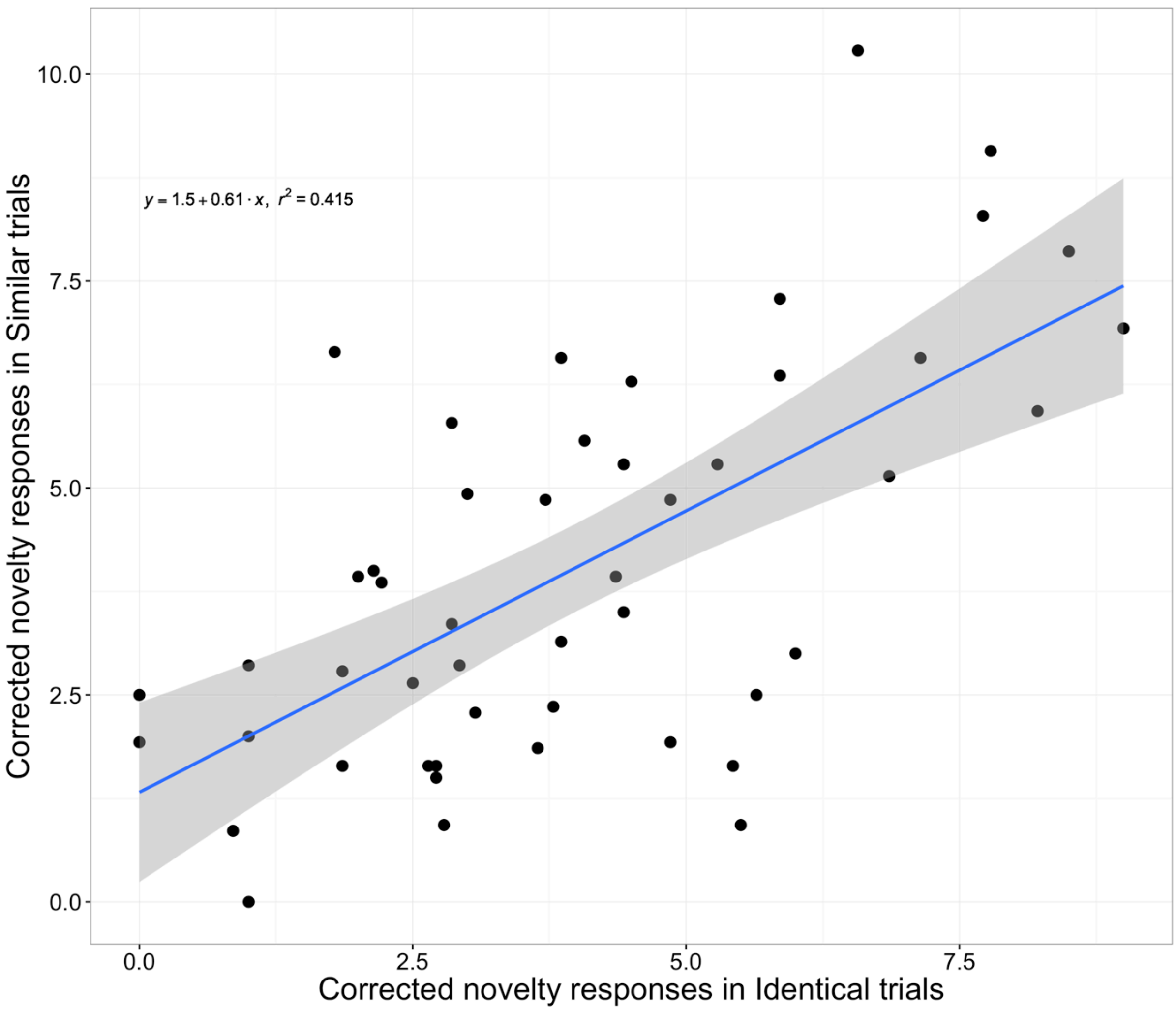


**Figure S7**. The relationship of novelty responses in 'Identical' and 'Similar' trials; blue line indicates assumed linear fit; grey area denotes 95% confidence interval.
